# Non-linear Min protein interactions generate harmonics that signal mid-cell division in *Escherichia coli*

**DOI:** 10.1371/journal.pone.0185947

**Published:** 2017-10-17

**Authors:** James C. Walsh, Christopher N. Angstmann, Iain G. Duggin, Paul M. G. Curmi

**Affiliations:** 1 School of Physics, University of New South Wales, Sydney, NSW, Australia; 2 The Ithree Institute, University of Technology Sydney, Sydney, NSW, Australia; 3 School of Mathematics and Statistics, University of New South Wales, Sydney, NSW, Australia; Purdue University, UNITED STATES

## Abstract

The Min protein system creates a dynamic spatial pattern in *Escherichia coli* cells where the proteins MinD and MinE oscillate from pole to pole. MinD positions MinC, an inhibitor of FtsZ ring formation, contributing to the mid-cell localization of cell division. In this paper, Fourier analysis is used to decompose experimental and model MinD spatial distributions into time-dependent harmonic components. In both experiment and model, the second harmonic component is responsible for producing a mid-cell minimum in MinD concentration. The features of this harmonic are robust in both experiment and model. Fourier analysis reveals a close correspondence between the time-dependent behaviour of the harmonic components in the experimental data and model. Given this, each molecular species in the model was analysed individually. This analysis revealed that membrane-bound MinD dimer shows the mid-cell minimum with the highest contrast when averaged over time, carrying the strongest signal for positioning the cell division ring. This concurs with previous data showing that the MinD dimer binds to MinC inhibiting FtsZ ring formation. These results show that non-linear interactions of Min proteins are essential for producing the mid-cell positioning signal via the generation of second-order harmonic components in the time-dependent spatial protein distribution.

## Introduction

Bacterial cell division requires the precise placement and timing of the FtsZ division ring to produce two viable daughter cells. This involves a number of regulatory systems, which are still being identified [1, 2]. To date, two separate negative regulators of the location of the FtsZ division ring in *Escherichia coli* have been identified: nucleoid occlusion and the Min system [3]. The Min system prevents ring formation at the poles of the rod-shaped cells by locally inhibiting polymerization of FtsZ, which is essential for assembly of the cell division apparatus [4, 5]. To achieve this, the Min proteins (MinD and MinE) form an oscillatory spatiotemporal pattern. This can be seen as mobile concentration gradients of MinD originating alternately from the poles [6]. Inhibition of the Min protein system leads to a proportion of cells dividing near the cell poles [3], with subsequent formation of mini-cells [6].

Several studies have modelled the Min system using reaction-diffusion equations [7–10]. A partial differential equation model, based on the known molecular interactions of the Min proteins, accurately reproduces the spatiotemporal Min oscillations observed in cells [11]. These patterns are an example of “Turing patterns”, as postulated by Alan Turing in his landmark paper [12]. Within the Turing framework, non-linear reactions produce spatiotemporal patterning of the underlying proteins if the homogeneous steady-state protein concentrations are destabilized by the introduction of diffusion. Consistent with Turing patterns [13], the wild-type patterning of the Min system is dominated by a single first-order cosine distribution of protein concentration along the long axis of the cell, which alternates over time between the two poles of the cell [13–16].

The generally accepted description of how the Min system functions is that the oscillating, polar localization of the Min proteins results in a Min-depleted zone towards the centre of the cell, allowing FtsZ assembly and cell division in this region [17]. Indeed, time-averaging microscopy images of fluorescently-labelled Min proteins shows a zone of reduced Min protein concentration around mid-cell [14–16]. However, the oscillating first-order cosine wave, which dominates the distribution of protein concentration in wild-type patterning [13], does not produce a mid-cell signal (MinD-depleted zone) when averaged over time. To produce the robust mid-cell signal seen *in vivo*, higher-order harmonic terms are likely to play a role. These harmonics are a natural consequence of the non-linear reactions seen in the Min system.

In this paper, we report a spatial Fourier spectral analysis of the Min system to investigate the role of higher-order harmonics in creating a minimum in the protein concentration at the mid-cell region when the time-average of the oscillating protein distribution is taken. From this analysis, the second-order cosine wave was identified as the key harmonic component that can give rise to the mid-cell minimum. The characteristics of the second-order Fourier component for the MinD distribution were identified by examining previous published experimental measurements of a fluorescently-labelled Min system [14]. Similar analysis was then performed on the MinD dynamics of the theoretical molecular model [11], confirming that the same characteristics are present. Fourier analysis was then performed on the individual molecular species (MinD monomer, dimer, etc.) of the Min model. This indicated that the Min system optimizes the mid-cell minimum of the membrane-bound MinD dimer species. The dynamics are such that the MinD dimer not only has the greatest contrast in the mid-cell minimum but also the most consistent signal over time. This result appears to be biologically relevant as the membrane-bound MinD dimer binds MinC that acts as an inhibitor of the assembly of the cell division machinery.

## Materials and methods

### Experimental kymograph analysis

The kymographs analysed in this work were created by integrating the fluorescent signal over a fixed distance perpendicular to the major axis of the cell [14]. At the poles of the cell, the diameter of the cell perpendicular to the major axis decreases because of the rounded end caps. As a result, the perpendicular fluorescence line integral effectively occurs over a shorter length in comparison to the mid-cell. Subsequently, the same concentration of fluorescent protein has a lower value in the kymograph at the poles than in the cell centre. To prevent the decrease in cell fluorescence due to the end caps from skewing the analysis, the last 5% of the cell length at each cap was excluded.

In this work, the experimental kymographs and cell lengths at the start and end of measurements originate from the studies of Fischer-Friedrich *et al*., which used fluorescently-labelled MinD-GFP [14]. To determine the position of the ends of the cell at each time point, the length of the cell was linearly interpolated between the length of the cell at the start and end of an experiment. At each time point, the region in the spatial direction of the kymograph equal to the length cell containing the maximum summation of fluorescent intensity was determined. A fit of these end points as a function of time resulted in two straight lines correlating to the ends of the cell as a function of time for the duration of the kymograph.

Fourier analysis was performed on the trimmed kymograph (i.e. last 5% of each end cap removed) using Mathematica 10.2 (Wolfram Research). In particular, the FourierDCT function was used at each time point in the kymograph to calculate the discrete Fourier cosine transform of the spatial fluorescence distribution along the cell axis. The resulting Fourier coefficients are equal to those obtained by the normalised Fourier cosine series

$$c_{n}=\frac{\int_{0}^{l}f(x)\cos(\frac{n\pi }{l}x)dx}{\int_{0}^{l}\cos^{2}(\frac{n\pi }{l}x)dx}$$ (1)

where *c*_*n*_ is the *n*th order Fourier coefficient for the basis set, with *l* as the length of the cell. This basis decomposition is equivalent to performing a Fourier transform on the distribution which is first reflected at the end of the cell (*f*(*x*) = *f*(2*l–x*) for *l* < *x* < 2*l*) before being made periodic (*f*(*x*) = *f*(*x* mod 2*l*)).

The temporal average of experimental data was formed by resampling the kymograph so that each time point contained 50 pixels along the length of the cell. Each respective pixel was then averaged over time to form the temporally averaged distribution.

To calculate the time-average spatial protein distribution due to the second-order Fourier component, the mean Fourier coefficient of the second-order cosine wave in the respective data provided the amplitude for a second-order cosine wave. This wave was added to the homogenous mean concentration of MinD (the concentration if no patterning occurred) to illustrate the extent of mid-cell signalling provided by the second-order mode. This resulted in the formula $\bar{c_{0}(t)}+\bar{c_{2}(t)}\cos(\frac{2\pi }{l}x)$, where $\bar{c_{0}(t)}$ is the mean zero-order Fourier coefficient (the mean concentration) and $\bar{c_{2}(t)}$ is the mean Fourier coefficient of the second-order mode.

### Theoretical model

The model of the Min system used in this work is adapted from a model previously proposed by Walsh *et al*. [11]. This model contains six species: MinD monomer in solution (*D*), MinE homodimer in solution (*E*_*2*_), membrane-bound MinE homodimer (*e*_*2*_), membrane-bound MinD monomer (*d*), membrane-bound MinD dimer (*d*_2_), and a membrane-bound MinDE heterotetramer complex (*d*_*2*_*e*_*2*_).

This molecular model is based on the experimentally identified interactions of the Min proteins. In particular, crystal structures have shown that MinD monomers (*d*) are able to form dimers (*d*_*2*_) in an ATP dependent manner [18]. Furthermore, FRET and yeast two hybrid experiments have shown that this occurs in a two-step process. MinD monomers in solution (*D*) first bind to the membrane as a monomer (*d*) before forming a dimer while bound to the membrane (*d*_*2*_) [19, 20]. MinE is a stable homodimer which undergoes a large-scale structural change between its inactive cytosolic and active membrane-bound forms [21–24]. NMR experiments have shown that MinE heavily favours its inactive cytosolic form (*E*_*2*_) [23] unless it is stabilized in its active conformation by binding to a membrane-bound MinD dimer to form a MinDE complex (*d*_*2*_*e*_*2*_) [21]. While in this complex, MinE allosterically activates MinD to hydrolyse ATP [25] causing the complex to dissociate with MinD being released into the cytosol as monomers (*D*) while MinE returns to being an unstable membrane-bound homodimer (*e*_*2*_).

In order for the molecular model of the Min system to reproduce the experimental kymographs, it was hypothesized that membrane-bound MinD monomer (*d*) can be removed from the membrane by interacting with membrane-bound MinE (*e*_*2*_). The inclusion of this interaction allows the model to recreate many of the characteristics of Min patterning seen *in vivo* [11].

**Fig 1:**
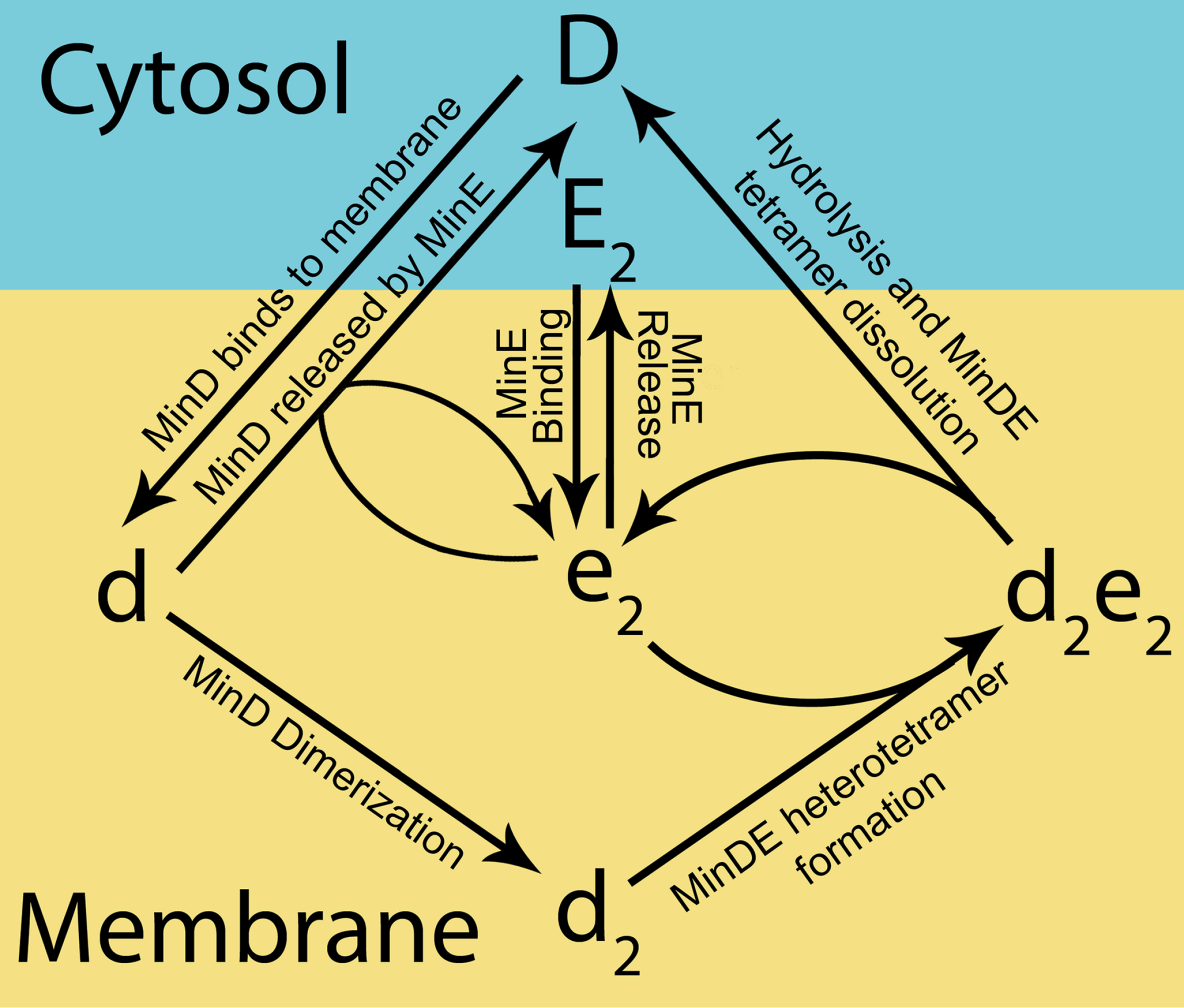
Overview of the simplified reactions underlying the model. A schematic showing the different states of the system and the reaction pathways between them. Cytosolic components (MinD monomer and MinE dimer) are represented by upper case letters (*D* and *E*_*2*_, respectively), while membrane-bound species (MinE dimer, MinD monomer, dimer and the MinDE heterotetramer) are represented by lower case letters (*e*_*2*_, *d*, *d*_*2*_, and *d*_*2*_*e*_*2*_, respectively). The value used for each of these parameters and a description of each reaction is given in Table 1. These parameter values are the same as used by Walsh *et al*. [11].

These experimentally determined properties of the Min proteins are incorporated into the model of the Min system by the following reactions: MinD monomer in solution (*D*) can bind to the membrane to give rise to a membrane-bound MinD monomer (*d*). The membrane-bound MinD monomer (*d*) can either form a dimer (*d*_*2*_), or be released from the membrane when the latter is stimulated by the membrane-bound MinE homodimer (*e*_*2*_). The MinD dimer (*d*_*2*_) can bind to the membrane-bound MinE (*e*_*2*_) to form a MinDE heterotetramer complex (*d*_*2*_*e*_*2*_). Following ATP hydrolysis, this complex dissociates resulting in MinD being released into the cytosol as monomers (*D*) while MinE returns to being an unstable membrane-bound homodimer (*e*_*2*_). MinE dimer in solution (*E*_*2*_) is able to bind to the membrane to give rise to the membrane-bound homodimer (*e*_*2*_) which, in turn, can be released back into the cytosol. These simplified reactions are shown schematically in Fig 1.

To simplify the analysis, we assume that the shape of the *E*. *coli* cell can be approximated by a cylinder of constant radius and length *l(t)*. Following this, the model was further simplified and collapsed to one dimension by making the assumption that the radial concentrations of soluble species (*D* and *E*_*2*_) are homogeneous. To make one-dimensional simulations approximately equivalent to a cylindrical domain with radius *R*, interactions between species in solution and the membrane (binding and release reactions) cause the concentration of the species in solution to change by the factor *2/R* (equal to the surface to volume ratio) greater than the rate of change of the membrane-bound species.

In growing domain simulations, we assume that the cells are grown at a constant rate *g*, so that *l*(*t*) = *l*_0_ + *gt*. The equations are solved on a fixed length domain with the growth incorporated by scaling the diffusion constants by a factor of $\frac{l_{0}^{2}}{{\left(l_{0}+gt\right)}^{2}}$ where *l*_*o*_ is the initial length of the cell [26]. Zero flux boundary conditions are taken at the two end points of the domain to ensure conservation of mass.

To conserve local protein number, a decay term proportional to the respective species, taking the form of $\frac{-g}{l_{0}+gt}$ was added to each species in the system as described in [26]. This counteracts the creation of proteins resulting from the rescaling of diffusion. To maintain a constant average concentration of protein, the expression of MinD and MinE are added through homogenous production of cytoplasmic MinD (*D*) and MinE (*E*_*2*_). These terms take the form of $\frac{gN_{D}}{l_{0}+gt}$ and $\frac{gN_{E}}{l_{0}+gt}$ where *N*_*D*_ (1389 μm^-3^) and *N*_*E*_ (486 μm^-3^) are the number concentrations (with units of number of proteins per cubic micron) of MinD monomer units and MinE dimer units, respectively.

The final equations used in this paper were thus:

$$\begin{array}{l} \partial_{t}D=\frac{2}{R}\left(\omega_{edf}e_{2}\cdot d+2\omega_{tetd}d_{2}e_{2}-\omega_{db}D\right)+\frac{g}{l_{0}+gt}\left(N_{d}-D\right)+\frac{l_{0}^{2}D_{D}}{{\left(l_{0}+gt\right)}^{2}}\partial_{z}^{2}D\\ \partial_{t}E_{2}=\frac{2}{R}\left(\omega_{er}e_{2}-\omega_{eb}E_{2}\right)+\frac{g}{l_{0}+gt}\left(N_{e}-E\right)+\frac{l_{0}^{2}D_{E}}{{\left(l_{0}+gt\right)}^{2}}\partial_{z}^{2}E_{2}\\ \partial_{t}d=\omega_{db}D-2\omega_{dim}d^{2}-\omega_{edf}e_{2}\cdot d-\frac{gd}{l_{0}+gt}+\frac{l_{0}^{2}D_{m}}{{\left(l_{0}+gt\right)}^{2}}\partial_{z}^{2}d\\ \partial_{t}d_{2}=\omega_{dim}d^{2}-\omega_{edf}e_{2}\cdot d_{2}-\frac{gd_{2}}{l_{0}+gt}+\frac{l_{0}^{2}D_{m}}{2{\left(l_{0}+gt\right)}^{2}}\partial_{z}^{2}d_{2}\\ \partial_{t}e_{2}=-\omega_{er}e_{2}+\omega_{eb}E_{2}-\omega_{edf}e_{2}\cdot d_{2}+\omega_{tetd}d_{2}e_{2}-\frac{ge_{2}}{l_{0}+gt}+\frac{l_{0}^{2}D_{m}}{2{\left(l_{0}+gt\right)}^{2}}\partial_{z}^{2}e_{2}\\ \partial_{t}d_{2}e_{2}=\omega_{edf}e_{2}\cdot d_{2}-\omega_{tetd}d_{2}e_{2}-\frac{gd_{2}e_{2}}{l_{0}+gt}+\frac{l_{0}^{2}D_{m}}{4{\left(l_{0}+gt\right)}^{2}}\partial_{z}^{2}d_{2}e_{2} \end{array}$$ (2)

The parameters used for simulations can be found in Table 1 and they are the same as those used by Walsh *et al*. [11].

**Table 1:** Rate parameters and reaction summary.

Rate	Reaction	Value	Units
ω_*db*_	MinD binding to membrane	4	μm.s^-1^
ω_*edf*_	MinD-MinE complex formation	22	μm^2^.s^-1^
ω_*dim*_	MinD dimerization	0.002	μm^2^.s^-1^
ω_*tetd*_	ATP hydrolysis & Tetramer dissociation	0.12	s^-1^
ω_*eb*_	MinE binding to membrane	0.07	μm.s^-1^
ω_*er*_	MinE release from membrane	30	s^-1^
D_*D*_	Diffusion of labelled MinD in solution	16	μm^2^.s^-1^
D_*E*_	Diffusion of MinE in solution	20	μm^2^.s^-1^
D_m_	Diffusion of membrane-bound states	0.1	μm^2^.s^-1^
*N*_*D*_	Total concentration of MinD monomer units	1389	μm^-3^
*N*_*E*_	Total concentration of MinE dimer units	486	μm^-3^
*g*	Growth rate of cell	0.625	nm.s^-1^

The one spatial dimension nonlinear PDE model was solved using Mathematica by employing the method of lines [27]. A structured point centre mesh consisting of 100 points was used. Initial conditions were taken as the homogeneous steady state concentrations multiplied by a uniformly distributed random value between 0.95 and 1.05 at each node point of the mesh.

Fourier analysis was performed on the solution to the PDEs in the same manner as per the experimental data.

To quantitatively analyse the harmonic components of the different species of the Min system, the maxima of the total MinD distribution pattern in a cell of length of 3.5 μm were identified. From these points, a time window corresponding a single oscillation (peak to peak) of the dominant first-order mode was identified. Within this window, the critical points were found (maxima, minima etc.) and measured. The amplitude was taken as the maximum of the amplitude minus the minimum divided by two. The phase measurements were normalized to span from zero to one. To convert to radians, phase values should be multiplied by a value of 2π, or to convert to seconds should be multiplied by the period of oscillation (88 s).

## Results

**Fig 2:**
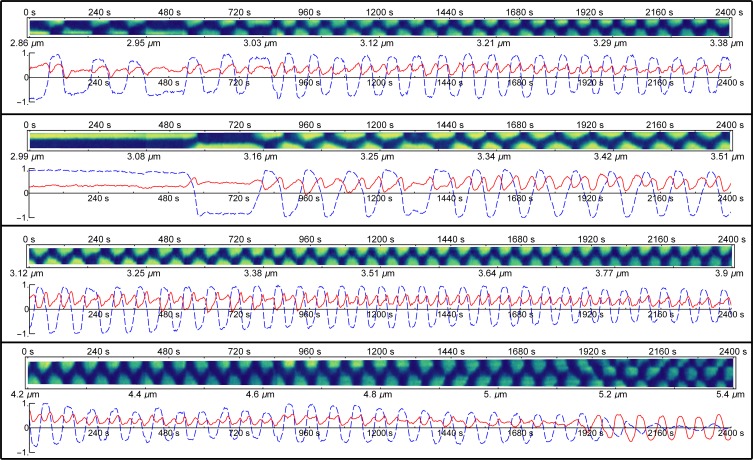
Fourier analysis of experimental data. Within each example the top panel shows an experimental kymograph. For each kymograph the main axis of the cell is plotted on the y axis with time plotted on the x axis. High MinD concentration is coloured in yellow while low MinD concentration is dark blue. The lower panel of each example shows the corresponding Fourier analysis of the kymograph, that is, a plot of the two lowest inhomogeneous terms of the spatial discrete cosine transform which is calculated at each time step of the kymograph. The first-order mode is shown in the dashed blue line while the second-order mode is shown in the solid red line.

**Fig 3:**
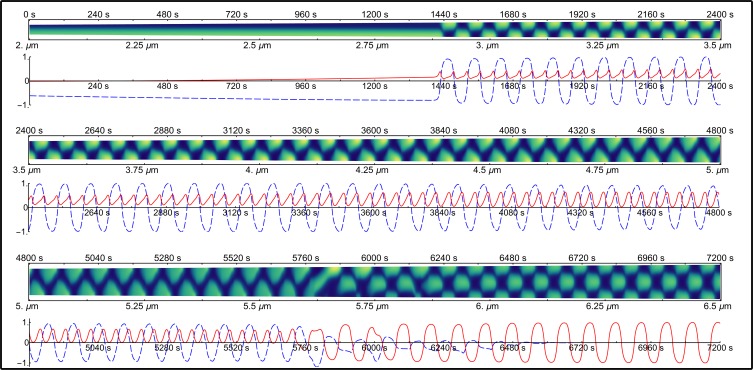
Fourier analysis of the single growing domain simulation. The cell grows from 2 to 6.5 μm over the course of 7200 seconds. Below each line of kymograph, the corresponding Fourier analysis is presented. The first-order mode is shown in the dashed blue line while the second-order mode is shown in the solid red line.

In rod-shaped *E*. *coli*, the distribution of Min proteins is effectively a function of the position along the major axis of the cell. Experimental studies have utilized kymographs to represent the spatial distribution of fluorescently-labelled Min proteins. To do this, the fluorescence intensity from a cell is collapsed to a line running along the major axis of the cell by integrating the signal perpendicular to it. This one-dimensional line is then plotted against time to create a kymograph. Samples of experimental kymographs of MinD-GFP fluorescence which originate from the studies of Fischer-Friedrich [14] are shown in Fig 2. In Fig 3, we present the distributions of MinD resulting from the model simulations. In both experimental and calculated kymographs, yellow (light regions) represents high protein concentration and blue (dark regions) represents low concentration.

In this paper, we use Fourier analysis to decompose the spatial distribution of protein observed in the kymographs at each time point. A Fourier cosine transform was used, producing a series of Fourier coefficients, each of which varies with time as the patterning evolves. The zero-order Fourier coefficient is a measure of the mean concentration of the protein species as measured by average fluorescence. The first-order Fourier coefficient represents the spatial variation in fluorescence or protein concentration that follows a half period cosine function with a maximum at one end of the cell, a node at mid-cell and a minimum at the opposite pole. The second-order Fourier coefficient is the next component, which follows a full cosinusoidal variation with either maxima at the poles and a mid-cell minimum or minima at the poles and a maximum at mid-cell.

As cells grow, spatial patterning changes. For normal (short) cells, patterning of the Min proteins is dominated by the first-order Fourier coefficient (*i*.*e*. the Min proteins oscillate from pole to pole and are hence described by a cosine distribution plus a constant). We refer to this as “first-order patterning”. When cells grow longer (to a point where normal cells would usually divide), the patterning changes to one where the spatial protein distribution is dominated by the second-order Fourier coefficient (*i*.*e*. the Min proteins alternate between being concentrated simultaneously at the two poles and then at mid-cell). We refer to this as “second-order patterning”.

Below each kymograph is a plot showing the time-dependent magnitude of the Fourier coefficients for the lowest two discrete spatial cosine transform terms (Figs 2 and 3). For the Fourier coefficient of the first-order cosine mode (dashed blue line), positive values represent a maximum at the top of the cell and a minimum at the bottom of the cell, where “top” and “bottom” refer to the two poles of the cell as it is aligned in the kymograph. Conversely, a negative value represents a cosine wave with low concentration at the top and high concentration at the bottom. For the Fourier coefficient of the second-order cosine mode (solid red line), positive values correspond to a second-order cosine distribution with maxima at both poles of the cell and a minimum at mid-cell, whereas negative values correspond to a single maximum at mid-cell with two minima—one at each pole.

### Time dependence of the spatial Fourier components for the MinD distribution

#### Time-dependent behaviour of the second-order Fourier coefficient is robust during first-order patterning

Representative examples in Fig 2 show the large variations in the patterning of fluorescently-labelled MinD that are seen across the experimental data [14]. Variations are seen in the length of the cell at which the MinD distribution makes a transition from a stationary to an oscillating pattern. This is demonstrated in the first two kymographs in Fig 2. In the second kymograph, the transition to an oscillating pattern occurs at 3.16 μm, which is a greater cell length than in the first kymograph where the system oscillates irregularly from the start of the experiment (2.86 μm) and becomes regular at 3.03 μm. Furthermore, once stable oscillations begin, the period of oscillation is much longer (130 s) in the second kymograph compared to the first (86 s). Finally, the wave shape appears very triangular in the second kymograph compared to the rectangular patterning in the first kymograph.

Despite the large variations in the observed MinD patterning, the time-dependent characteristics of the second-order Fourier coefficient are robust (Fig 2, solid red line). In particular, the second-order Fourier coefficient is essentially always positive, oscillating with half the period of the first-order Fourier coefficient (Fig 2, dashed blue line). The oscillation of the second-order Fourier coefficient is approximately out of phase with that of the first-order Fourier coefficient, that is, the maxima of the second-order term approximately line up with time points where the first-order term is zero.

As shown in the third and fourth examples in Fig 2, this pattern continues throughout the growth of the cell until the cell begins to transition to second-order patterning, that is, patterning dominated by the second-order Fourier coefficient (5.2 μm in the fourth kymograph of Fig 2). This is the point at which a normal cell would begin to divide [14]. When the transition to second-order patterning occurs, the first-order Fourier coefficient (dashed blue line) decays in amplitude. The time-dependent behaviour of the second-order Fourier coefficient makes a transition from the positively offset, frequency-doubled patterning seen in shorter cells to an oscillation with much greater amplitude that oscillates at the same period as the original first-order Fourier coefficient and is centred around zero (i.e. it is no longer positively offset thus it corresponds to an alternation between maxima at the two poles of the cell and a maximum at mid-cell as seem in the fourth kymograph in Fig 2 for cell lengths greater than 5.2 μm).

The examples shown in Fig 2 are representative of the large collection of experimental kymographs for fluorescently-labelled MinD that have been analysed. Additional examples are presented in the Supporting Information (S1 Fig).

#### The model of the Min system reproduces dynamics of the second-order Fourier component

Fig 3 shows the distribution of MinD resulting from a full simulation of the Min model [11] that is solved on a one dimensional line using Eq 2. The key features of the time variation in the second-order Fourier coefficient (solid red line) described for the experimental data (Fig 2) were also present in the distribution of MinD calculated from the model (Fig 3). Namely, the second-order Fourier coefficient (solid red line) was positive, periodic with half the period of the first-order Fourier coefficient (dashed blue line) and the two Fourier terms were out of phase such that the maxima of the second-order Fourier coefficient approximately coincide with time points where the first-order Fourier coefficient was zero.

As per the experimental data, the dynamics of the second-order Fourier mode for the model continued throughout the growth of the cell until the cell began to transition to a second-order patterning (patterning dominated by the second-order component as seen in the third kymograph in Fig 3 at a cell length of 5.75 μm). The transition to second-order patterning occurred in a similar manner to the experimental data. At a critical length, the second-order Fourier coefficient became the dominant term and shifted to an oscillation with much greater amplitude, centred on zero with a period that was similar to that of the original first-order mode (*i*.*e*. a doubling of the period of the second-order Fourier coefficient) (Fig 3, third kymograph at 5.75 μm cell length).

#### Mid-cell minimum in the temporal average of the MinD concentration results from the second-order spatial Fourier component

**Fig 4:**
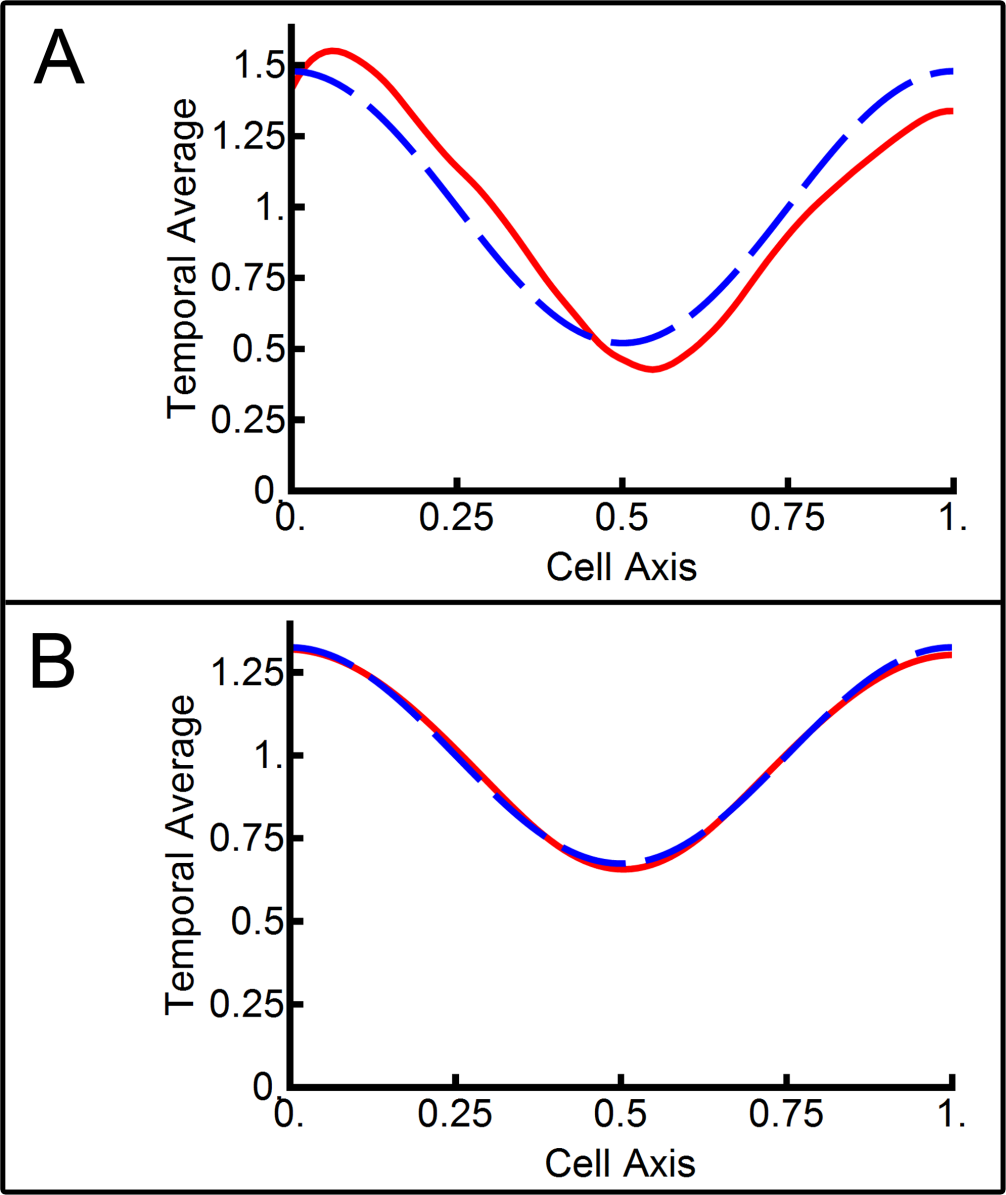
Temporal averages of the Min protein system. The temporal average of total MinD as a function of position along the cell axis is shown in the dashed blue line. The contribution of the second-order mode to the mid-cell minima is shown in the solid red line. **(A)** The temporal average of the third experimental kymograph from Fig 2. **(B)** The temporal average of the middle line of simulation from Fig 3.

The positioning of the FtsZ cell division ring is thought to occur, in part, because the oscillating MinD distribution leaves a bare zone in the middle of the cell that is depleted in MinD and thus depleted in its associated cell division inhibitor MinC [8]. To determine the source of the mid-cell minimum in MinD concentration, the temporal average of the third experimental kymograph in Fig 2 was taken. This example was chosen as the Min system exhibited stable, oscillating first-order patterning during this time. This avoided the temporal average being skewed by stationary patterning (seen in the first two kymographs in Fig 2) or the second-order patterning (seen in the last kymograph). The resulting temporal average of MinD-GFP fluorescence intensity is shown in the dashed blue line in Fig 4A. A clear mid-cell minimum was evident with a factor of three between the mid-cell MinD concentration and the concentration at the poles (assuming MinD-GFP fluorescence was proportional to MinD concentration).

The time-dependent behaviour of the first-order spatial Fourier coefficient for the MinD distribution showed an oscillation that was symmetric about zero (Fig 2, dashed blue line). Thus, its temporal average was zero and it subsequently did not contribute to the observed mid-cell minimum in time-averaged MinD concentration, *i*.*e*. its time-average resulted in a homogeneous spatial distribution of MinD. In contrast, the second-order Fourier coefficient in MinD spatial distribution remained positive throughout first-order patterning (Fig 2, solid red line). Thus, it contributed to a non-homogeneous time-averaged spatial distribution. The contribution of the second-order Fourier component to the time-averaged MinD spatial distribution is shown in the solid red curve in Fig 4A.

The strong correlation between the experimental temporal-average MinD concentration (Fig 4A, dashed blue line) and the mid-cell minimum resulting from the second-order Fourier term (Fig 4A, solid red line) indicated that the second-order spatial Fourier component was responsible for the temporally averaged mid-cell minimum in MinD protein concentration.

The equivalent plot of the temporal average of total MinD (dashed blue curve) and second-order Fourier component contribution (solid red curve) calculated from a simulation of the Min model is shown in Fig 4B. The data used for this analysis comes from the central kymograph in Fig 3. Again, this was chosen as it was free of stationary and second-order patterning (Fig 3, top and bottom kymographs, respectively). As with the experimental data, the strong correlation between the temporal average of total MinD concentration (Fig 4B, dashed blue line) and the second-order Fourier component contribution (Fig 4B, solid red line) shows that the second-order spatial Fourier component is essentially solely responsible for the temporally averaged mid-cell minimum in MinD concentration produced by the model.

While the higher-order Fourier coefficients of the spatial MinD concentration were non-zero, their impact on the function of the Min system (i.e. positioning of FtsZ) during first-order patterning was likely to be small given that the second-order Fourier component essentially generates the mid-cell minimum in MinD concentration on temporal averaging. Despite this, the higher-order Fourier coefficients still provide a means to test the accuracy of any model. The time dependence of these higher-order spatial Fourier coefficients for MinD concentration obtained from experimental kymographs (S2 Fig) and the Min model (S3 Fig) are presented in the Supporting Information section "Higher-order mode analysis". The correspondence between the temporal behaviour of these higher-order Fourier coefficients that were obtained from the experimental data (S2 Fig) and the model (S3 Fig) is testimony to the ability of our model to represent the experimental data.

### Time dependence of the spatial Fourier coefficients describing the individual protein components of the Min system

#### Analysis of the behaviour of individual components of the Min system

**Fig 5:**
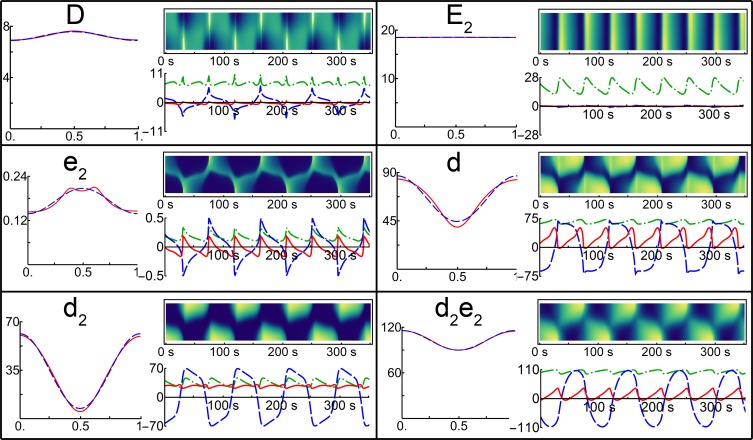
Fourier analysis of individual species. Kymographs and corresponding Fourier analysis for the individual species of the Min model when simulated with a cell of length 3.5 μm for 350 s are shown on the right-hand side of each panel. Under each kymograph the dashed blue line shows the first-order mode, the solid red line the second-order mode and the dot-dashed green line represents the zero-order mode (the temporal variation in concentration). The temporal average of each species as a function of position along the cell axis is shown on the left-hand side of each panel in the dashed blue line with the second-order mode contribution shown in the solid red line.

The ability of the Min model to accurately reproduce the experimentally measured higher-order harmonics (spatial Fourier components) of total MinD concentration suggests that the model may also accurately reproduce the higher-order Fourier coefficient dynamics on the level of individual component species of the Min system. The same Fourier analysis used for the total MinD concentration was applied to each individual component species of the Min system model. The results of this analysis are shown in Fig 5 and Table 2. The model simulations were solved on a fixed-sized cell of length 3.5 μm. Fourier analysis of complete growing cell simulations are provided in the Supporting Information (S4 Fig).

**Table 2:** Quantification of Fourier analysis.

	Units	D	E_2_	e_2_	d	d_2_	d_2_ e_2_
**Zero-order mean**	μm^-2^	7.24	18.53	0.17	65.59	34.42	102.8
**Zero-order amplitude**	μm^-2^	2.19	8.03	0.12	5.78	10.94	8.1
**Zero-order max. phase**	radians/2π	0.22	0.28	0.22	0.16	0.28	0.15
**First-order amplidude**	μm^-2^	6.54	0.92	0.5	68.71	69.44	106.6
**First-order max. phase**	radians/2π	0.22	0.22	0.22	0.73	0.79	0.07
**First-order node phase**	radians/2π	0.09	0.14	0.14	0.17	0.17	0.27
**Second-order mean**	μm^-2^	-0.34	-0.02	-0.03	21.11	26.98	12.73
**Second-order amplitude**	μm^-2^	0.68	0.08	0.18	26.58	4.17	22.74
**Second-order max. phase**	radians/2π	0.22	0.22	0.22	0.16	0.16	0.21

Table 2 presents the quantitative parameters obtained by Fourier analysis. The first row in Table 2 gives the mean of the zeroth order component of the Fourier transform, which is equivalent to the mean concentration in units of molecules per μm^2^, i.e. the concentration projected onto the cell membrane. To convert these surface concentrations to volumetric concentrations, one needs to multiply the numbers in Table 2 by the surface to volume ratio of the model cell (i.e. 4 μm^-1^).

Thus, the Fourier analysis gives the concentration of each species in the MinD-MinE system. From Table 2, 85% of MinE was present in the membrane-bound MinDE complex (*d*_*2*_*e*_*2*_) with 15% in the soluble MinE dimer (*E*_*2*_). Only 0.14% of MinE was present in the free, membrane-bound MinE dimer (*e*_*2*_). The majority of MinD (59%) was present in the MinDE complex (*d*_*2*_*e*_*2*_) with approximately equal portions in the remaining membrane-bound states monomer (*d*) and dimer (*d*_*2*_) with 19% and 20%, respectively. Only 2% of MinD was present as a monomer in solution (*D*). Table 2 also contains the amplitude (max value minus the min value divided by two) of the zero, first and second-order spatial Fourier coefficients for each species.

By examining the phase of the maximum of the dominant first-order mode graphically in the plot of the Fourier coefficient versus time in Fig 5 (dashed blue line) and quantitatively in Table 2, it is clear that the different species can be broadly clustered into two groups. The first-order mode of membrane-bound MinD monomer (*d*), dimer (*d*_*2*_) and the MinDE complex (*d*_*2*_*e*_*2*_) species were all approximately in phase with each other (with values of 0.73, 0.79 and 1.07 respectively). This result reflects that these species oscillated from pole to pole at the same time as can be seen in the kymographs (Fig 5). Phase measurements were normalized to span from zero to one. These values can be converted to radians be multiplying by 2π, or to converted to seconds by multiplying by the period of oscillation (88 s).

The remaining species, MinD (*D*) and MinE (*E*_*2*_) in solution, as well as the membrane-bound MinE homodimer (*e*_*2*_), also oscillated together, with all three species having a dominant first-order mode phase of 0.22. This value was approximately half a period less than the other three species (phase difference of 0.51 of a period between these three species and the MinD monomer (*d*)) so these species oscillated together, but did so out of phase relative to the other three species, *i*.*e*. when these species have a maximum at one pole, the other three species had a maximum at the opposite pole. Note that the time-averaged spatial pattern for MinE in solution was miniscule with the protein approximately uniformly distributed along the length of the cell (Fig 5, top right panel).

The time-dependent behaviour of the second-order Fourier coefficients also clustered the species into the same two groups based on whether they oscillate about zero or not. The second-order Fourier coefficients of the MinD monomer (*d*), dimer (*d*_*2*_) and the MinDE complex (*d*_*2*_*e*_*2*_) all oscillated in time with a positive offset, with mean values of 21.11 μm^-2^, 26.98 μm^-2^ and 12.73 μm^-2^, respectively. The mean values for the second-order Fourier coefficients of the remaining three states were small and negative, reflecting that the oscillations of their respective second-order modes were centred slightly below zero amplitude.

#### Min dynamics optimize the mid-cell minimum of the MinD dimer species

Each molecular species in the Min system displayed slightly different dynamics in the behaviour of the second-order spatial Fourier coefficient. To examine the potential role of each molecular species in defining the mid-cell division site, the temporal averages of each individual species were calculated (Fig 5, dashed blue curve in the left-hand side of each panel). The contribution of the second-order Fourier component to each temporal average is shown in the solid red curve (Fig 5, left-hand side of each panel). As with the MinD ensemble averages (Fig 4), the second-order Fourier component was responsible for essentially all of the mid-cell signal, be it a mid-cell minimum (*d*, *d*_*2*_ and *d*_*2*_*e*_*2*_) or maximum (*D* and *e*_*2*_).

The species showing the largest mid-cell minimum was the membrane-bound MinD dimer (*d*_*2*_). This species also had the greatest contrast between the mid-cell minimum and the maxima at the cell poles. Contrast is calculated from the difference between the maximum and minimum divided by their sum and is approximately equivalent to the ratio of the second-order mean divided by the zero-order mean in Table 2. The contrasts for the three molecular species showing mid-cell minima are 0.78, 0.32 and 0.12 for the MinD membrane-bound dimer (*d*_*2*_), the MinD membrane-bound monomer (*d*) and the MinDE complex (*d*_*2*_*e*_*2*_), respectively. Thus, the mid-cell minimum of the MinD monomer (*d*) is also pronounced, albeit to a lesser extent than for the MinD dimer (*d*_*2*_). The MinDE complex (*d*_*2*_*e*_*2*_) shows a less pronounced mid-cell minimum.

The remaining three molecular species all have slightly negative second-order Fourier coefficient mean values (MinD in solution (*D*) -0.34, MinE in solution (*E*_*2*_) -0.02 and membrane-bound MinE (*e*_*2*_) -0.03). As a result, the temporal average of each of these three states has a mid-cell maximum, albeit small for MinD in solution (*D*) and virtually non-existent for MinE in solution (*E*_*2*_).

## Discussion

Harmonic analysis of the spatial distribution of MinD in *E*. *coli* cells shows a stable and robust pattern in the time-evolution of the individual Fourier components. As expected for short, rod-shaped *E*. *coli* cells (with rounded ends), the dominant mode is well described by a one-dimensional first-order cosine wave [13]. However, this term does not encode positional information when time-averaged (as is true for all odd-order cosine terms in cells that are symmetric about the mid plane perpendicular to the cell axis). Instead, the second-order Fourier component of the MinD distribution provides the majority of the spatial information defining the mid-cell region where division is to occur (Figs 2–4). The generation of a minimum in MinD concentration in the mid-cell region is critical, because MinD recruits the FtsZ inhibitor, MinC, preventing unwanted assembly of FtsZ away from the mid-cell region [28].

The dynamics of this second-order Fourier component in MinD concentration are remarkably robust. Despite the large variations in MinD patterning observed in both experimental data and during the time-evolution of the model, the essential behaviour of the second-order Fourier component remains surprisingly unchanged. The ability of the Min system to function *in vivo* despite large perturbations resulting from things such as GFP-labelling, over-expression of the Min operon and changes in temperature is probably a result of the robustness of this second-order mode.

We observe a strong correlation between the time-evolution of the Fourier components obtained from kymographs of MinD-labelled cells and those obtained from our model [11]. This correlation is maintained as the cell grows through three distinct regimes: stationary patterning, oscillating first-order patterning and oscillating second-order patterning, where the latter occurs when a wild-type cell would normally be dividing. Both the model and experimental data show the same time-evolution for the first four Fourier components.

The ability of our molecular model [11] to reproduce the dynamic behaviour of the higher-order Fourier components for MinD patterning that are present in the analysis of experimental kymographs gives weight to the veracity of the molecular interactions underlying the model. Given that the generation of harmonics is specific to the dynamics of the system, this suggests that the molecular reactions incorporated in the model probably reflect reality *in vivo*. Furthermore, as the model kymographs show exceptional similarity to the experimental kymographs, one would expect the Fourier transforms of the kymographs also to be similar.

By carrying out Fourier analysis on the individual species in the model Min system, we show that the membrane-bound MinD dimer (*d*_*2*_) carries the strongest signal for mid-cell positioning. This molecular species shows the greatest contrast between the mid-cell minimum concentrations and the maxima at the poles when the protein concentration is temporally averaged (Fig 5). This arises because the second-order Fourier component of the MinD dimer (*d*_*2*_) has the largest relative positive offset. Additionally, the temporal dynamics of the second-order Fourier component of the MinD dimer (*d*_*2*_) show damped oscillations making the second-order Fourier coefficient essentially static in time (Fig 5). This is potentially important, since a stationary second-order Fourier component means that the mid-cell localization signal is constant. Thus, our analysis has shown that the membrane-bound MinD dimer not only has the strongest mid-cell signal but also the most stable signal.

The optimization of the mid-cell minimum in the spatial distribution of the membrane-bound MinD dimer (*d*_*2*_) is clearly utilized by *E*. *coli*. The dynamics of MinC is such that MinC is able to bind to MinD dimers but is then out competed by MinE so that MinC is not able to bind MinD when it is part of the membrane-bound MinDE complex [29]. Whether the copolymerization of MinC with membrane-bound MinD dimers then provides a further source of non-linear feedback to amplify the mid-cell MinD dimer signal remains an open question.

This work demonstrates that while Turing patterns in elongated cells are all likely to approximate cosine waves, the way in which the patterning is translated into a localization signal is likely to be specific to the dynamics of the system. In particular, it is specific to the higher-order harmonics generated by the non-linear molecular interactions of the system. This raises the question: what other higher-order mode dynamics are possible?

The second-order mode of the MinE homodimer (*e*_*2*_) (Fig 5) has a negative offset. This translates into a mid-cell maximum in the MinE homodimer when the pattern is time-averaged (Fig 5). Thus, a system displaying a negatively offset second-order mode such as MinE (*e*_*2*_) could produce a localization signal to position proteins to the cell poles (inhibiting localization at mid-cell) via an oscillatory Turing pattern.

## Conclusions

The Min system generates protein patterning dominated by a first-order Fourier component. This spatial first-order cosine distribution oscillates in time, moving Min proteins from pole to pole but producing no net spatial signal when averaged over time. Instead, the localization signal that identifies the mid-cell region is carried by the second-order Fourier component of the MinD distribution. This is a result of the non-linear dynamics of the Min system. In particular, the membrane-bound MinD dimer (*d*_*2*_) shows the localization signal with the highest contrast and the smallest time dependence. This appears to be biologically relevant as the MinD dimer (*d*_*2*_) binds to MinC, the protein that inhibits FtsZ ring formation. The molecular interactions underlying the Min system ensure that the dynamics observed for this second-order Fourier component are robust, helping to maintain a faithful positioning of the cell-division machinery.

## Supporting information

S1 File Supporting information. (PDF)
