# Supplementary material for: Non-linear Min protein interactions generate harmonics that signal mid-cell division in Escherichia coli
Source: PLoS One. 2017 Oct 17;12(10):e0185947. doi: 10.1371/journal.pone.0185947 (PMC5645087; doi:10.1371/journal.pone.0185947)
Supplement: S1 File — (PDF) [file pone.0185947.s001.pdf]

Non-linear Min protein interactions generate harmonics that signal mid-cell division in *Escherichia coli*

James C. Walsh[1,2], Christopher N. Angstmann[3], Iain G. Duggin[2,] & Paul M. G. Curmi[1,*]

Supporting Information

Additional Experimental Kymographs

A collection of experimental kymographs with their corresponding Fourier analyses are shown in Figure S1. Examples are ordered by their starting cell length. As a result most of the examples that start with stationary patterning and transition to oscillations are found in the first panel of Figure S1, while late stage phenomena including transitions to second order patterning are seen the second panel.

As with the representative examples in Figure 1, large variations in patterning are seen across the experimental data in Figure S1. Some examples oscillate despite being well below the mean critical length where oscillations begin. This is seen in the first example in Figure S1. Other examples do not oscillate despite being much larger than $2.7\mu m$. This is seen in the first example in the second panel of Figure S1. Furthermore, once stable oscillations begin the period of oscillation differs amongst samples.

Despite the large variations in the observed patterning, the characteristics of the second order mode (Figure S1, red line) during first order patterning are incredibly robust. In particular, the second order mode is essentially always positively offset, with double the frequency of the first order mode and it is approximately out of phase with the first order mode, that is, the maxima of the second order modes approximately lines up with where the first order mode is zero, similar to examples in the main text.

As shown by the examples in the second panel of Figure S1, the general characteristics of the second order mode continue throughout the growth of the cell until the cell begins to transition to a patterning regime dominated by the second order mode (called second order patterning). When this transition occurs (bottom three kymographs in the second panel of Figure S1), the first order mode (dashed blue line) decays in magnitude. The second order mode (red line) transitions from the positive offset, frequency double patterning seen in short cells and it is replaced by an oscillation with much greater amplitude which is centred around zero and has the same frequency as the original first order mode during first order patterning.

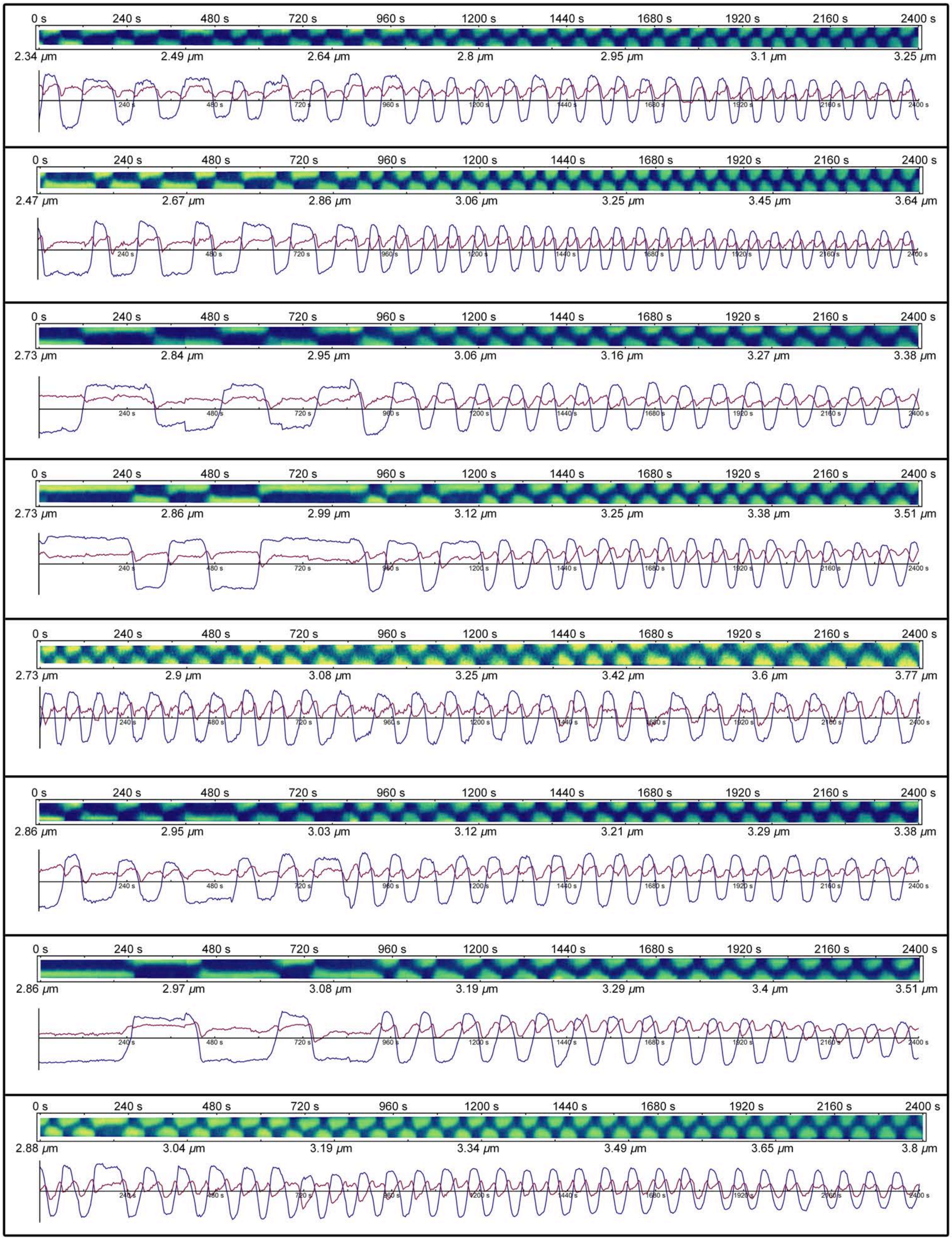
0 s 240 s 480 s 720 s 960 s 1200 s 1440 s 1680 s 1920 s 2160 s 2400 s
2.34 μm 2.49 μm 2.64 μm 2.8 μm 2.95 μm 3.1 μm 3.25 μm
2.47 μm 2.67 μm 2.86 μm 3.06 μm 3.25 μm 3.45 μm 3.64 μm
2.73 μm 2.84 μm 2.95 μm 3.06 μm 3.16 μm 3.27 μm 3.38 μm
2.73 μm 2.86 μm 2.99 μm 3.12 μm 3.25 μm 3.38 μm 3.51 μm
2.73 μm 2.9 μm 3.08 μm 3.25 μm 3.42 μm 3.6 μm 3.77 μm
2.86 μm 2.95 μm 3.03 μm 3.12 μm 3.21 μm 3.29 μm 3.38 μm
2.86 μm 2.97 μm 3.08 μm 3.19 μm 3.29 μm 3.4 μm 3.51 μm
2.88 μm 3.04 μm 3.19 μm 3.34 μm 3.49 μm 3.65 μm 3.8 μm

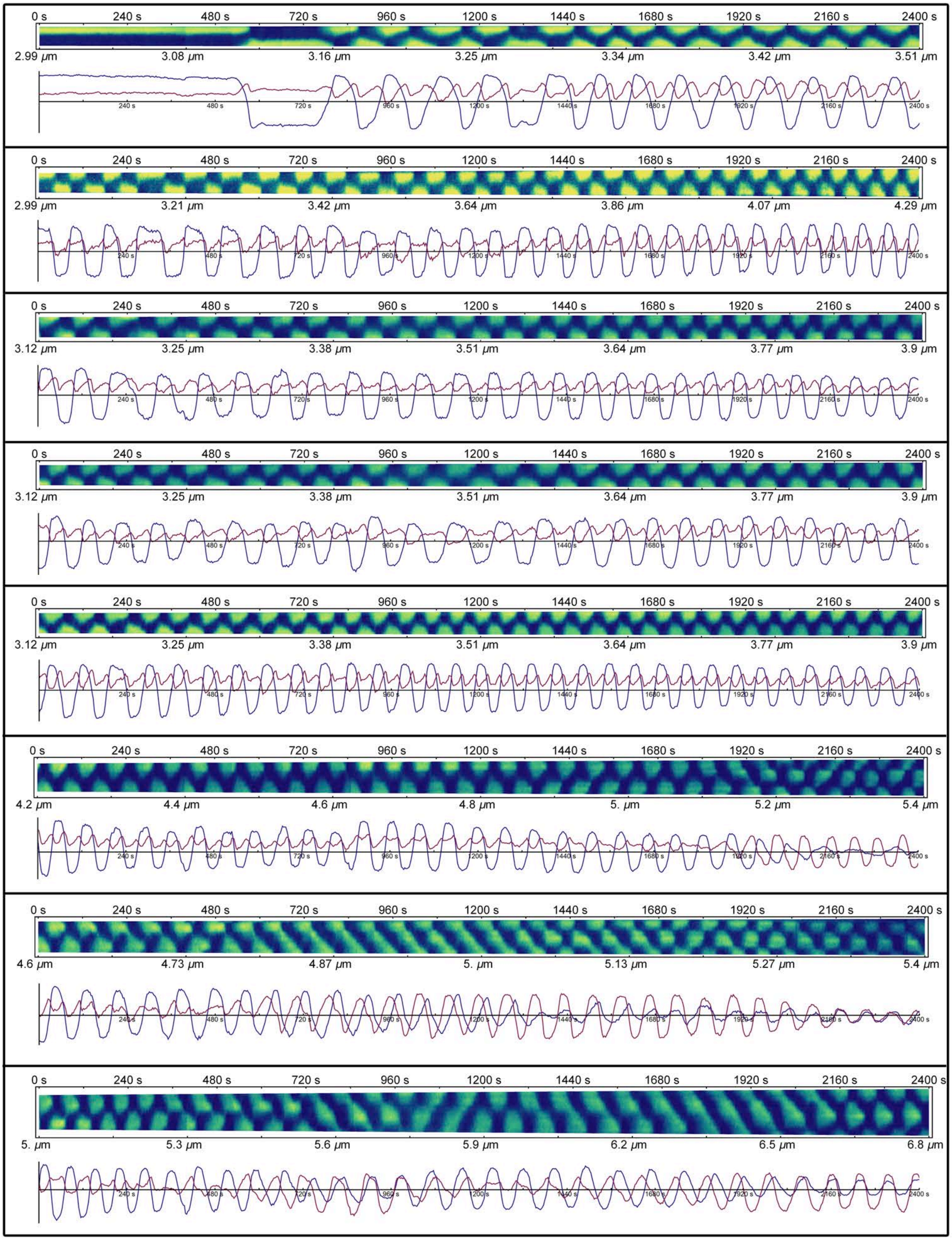
0 s 240 s 480 s 720 s 960 s 1200 s 1440 s 1680 s 1920 s 2160 s 2400 s
2.99 μm 3.08 μm 3.16 μm 3.25 μm 3.34 μm 3.42 μm 3.51 μm
0 s 240 s 480 s 720 s 960 s 1200 s 1440 s 1680 s 1920 s 2160 s 2400 s
2.99 μm 3.21 μm 3.42 μm 3.64 μm 3.86 μm 4.07 μm 4.29 μm
0 s 240 s 480 s 720 s 960 s 1200 s 1440 s 1680 s 1920 s 2160 s 2400 s
3.12 μm 3.25 μm 3.38 μm 3.51 μm 3.64 μm 3.77 μm 3.9 μm
0 s 240 s 480 s 720 s 960 s 1200 s 1440 s 1680 s 1920 s 2160 s 2400 s
3.12 μm 3.25 μm 3.38 μm 3.51 μm 3.64 μm 3.77 μm 3.9 μm
0 s 240 s 480 s 720 s 960 s 1200 s 1440 s 1680 s 1920 s 2160 s 2400 s
3.12 μm 3.25 μm 3.38 μm 3.51 μm 3.64 μm 3.77 μm 3.9 μm
0 s 240 s 480 s 720 s 960 s 1200 s 1440 s 1680 s 1920 s 2160 s 2400 s
4.2 μm 4.4 μm 4.6 μm 4.8 μm 5. μm 5.2 μm 5.4 μm
0 s 240 s 480 s 720 s 960 s 1200 s 1440 s 1680 s 1920 s 2160 s 2400 s
4.6 μm 4.73 μm 4.87 μm 5. μm 5.13 μm 5.27 μm 5.4 μm
0 s 240 s 480 s 720 s 960 s 1200 s 1440 s 1680 s 1920 s 2160 s 2400 s
5. μm 5.3 μm 5.6 μm 5.9 μm 6.2 μm 6.5 μm 6.8 μm

Figure S1: Fourier analysis of additional experimental kymographs. Within each example the top panel shows an experimental kymograph. For each kymograph the main axis of the cell is plotted on the y axis with time plotted on the x axis. High MinD is colored in yellow while low MinD concentration is dark blue. The lower panel of each example shows the corresponding Fourier analysis of the kymograph. That is, a plot of the amplitude of the first and second-order modes calculated using a spatial discrete cosine transform at each time step of the kymograph. The first-order mode is shown in blue while the second-order mode is shown in red.

Higher Order Mode Analysis of Experimental Data

The higher order mode dynamics of two representative experimental examples are shown in Figure S2. For each of the two examples, the relevant kymograph is shown at the top. Beneath this is a plot showing the four lowest terms of the spatial discrete cosine transform (excluding the zero order term) which is calculated at each time step of the kymograph. The first order mode is shown in blue, the second in red, the third in green and the fourth in gold. Beneath this are four plots, one for each of the four components, respectively, but with amplitudes of the plot scaled to allow for the characteristics of each mode to be identified.

Once regular oscillations begin (after 900 s in the first example), the first order mode (blue) oscillates in a regular manner centred on zero.

The second order mode (red) is positively offset, with double the frequency of the first order mode (blue) and it is out of phase such that the maxima of the second order mode approximately line up with where the first order mode is zero.

The third order mode (green) oscillates about zero and has the same period as the first order mode (blue) but in the top example, it is out of phase by approximately 180° with the first order mode such that a maximum in the first order mode aligns with a minimum in the third order mode and vice versa. In the second example, the oscillation of the third order mode is not as clear as in the top example.

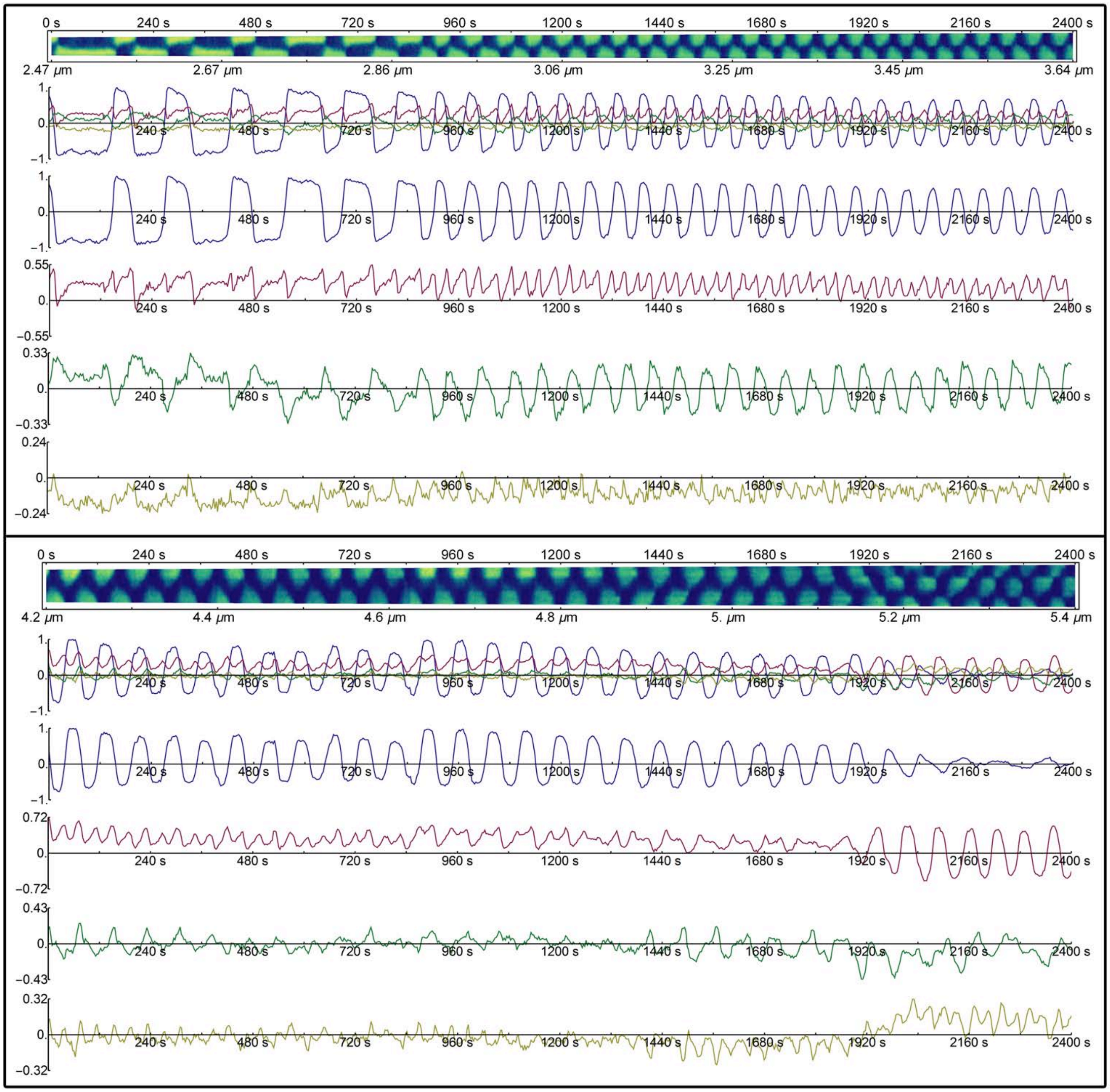

Figure S2: Higher-order mode analysis of experimental data. Two examples are shown, within each panel:(Top Line) Experimentally measured kymograph used for this analysis (Second Line) The overlay of the lowest four non-zero Fourier components to show relative magnitude (Third Line) The first-order mode shown in blue (Fourth Line) The second-order mode shown in red (Fifth Line) the third-order mode shown in green (Sixth Line) the fourth-order mode shown in green.

The fourth order mode (gold) appears to be approximately the negative of the second order mode (red). It is negatively offset with double the frequency of the first order mode (blue) and is out of phase with the second order mode (red) by about 180° such that maxima in the fourth order mode align with minima in the second order mode and vice versa.

Interestingly, when the second order mode becomes dominant toward the end of the second example (i.e. second order patterning), the fourth order mode assumes the characteristics of the second order mode in shorter cells, that is, positively offset with double the frequency and out of phase.

Higher Order Mode Analysis of Simulation

The higher order mode dynamics of MinD in the model simulation of a growing cell is shown in Figure S3. Similar to the experimental data in Figure S2, for each of the three panels of data, the relevant kymograph is shown at the top. Beneath this a plot showing the overlay of the four lowest order Fourier terms. The first order mode is shown in blue, the second in red, the third in green and the fourth in gold. Beneath this are four plots showing each of the four components separately.

The same general characteristics of the higher modes seen in the experimental data are also seen in the simulation.

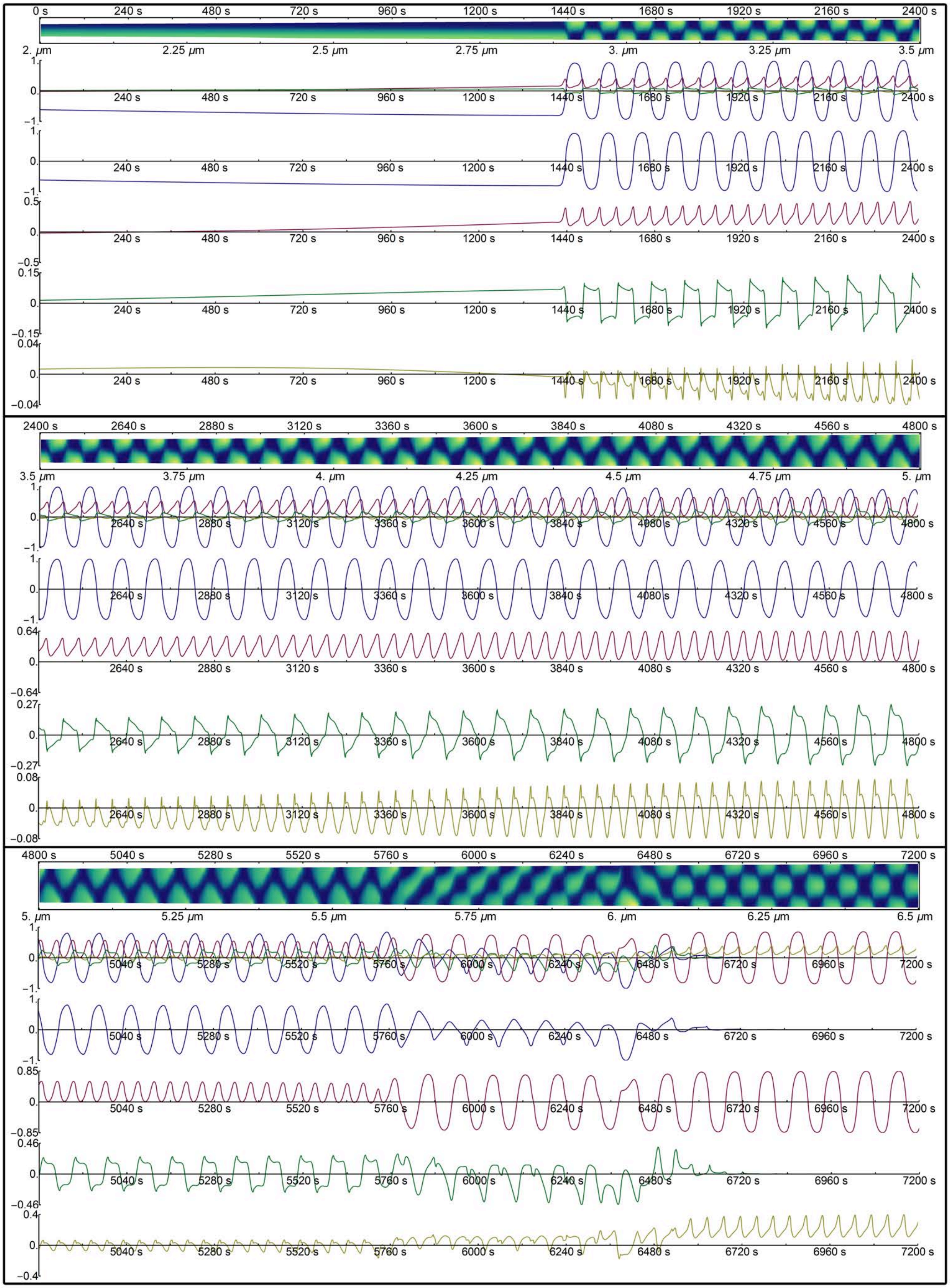

0 s 240 s 480 s 720 s 960 s 1200 s 1440 s 1680 s 1920 s 2160 s 2400 s
2. μm 2.25 μm 2.5 μm 2.75 μm 3. μm 3.25 μm 3.5 μm
1. 0. −1.
0.5 0. −0.5
0.15 0. −0.15
0.04 0. −0.04
2400 s 2640 s 2880 s 3120 s 3360 s 3600 s 3840 s 4080 s 4320 s 4560 s 4800 s
3.5 μm 3.75 μm 4. μm 4.25 μm 4.5 μm 4.75 μm 5. μm
0.64 0. −0.64
0.27 0. −0.27
0.08 0. −0.08
4800 s 5040 s 5280 s 5520 s 5760 s 6000 s 6240 s 6480 s 6720 s 6960 s 7200 s
5. μm 5.25 μm 5.5 μm 5.75 μm 6. μm 6.25 μm 6.5 μm
0.85 0. −0.85
0.46 0. −0.46
0.4 0. −0.4

Figure S3: Higher-order mode analysis of simulation. A single continuous simulation is divided into three parts that form the three panels. Within each panel: (Top Line) the kymograph resulting from the simulation; (Second Line) the overlay of the lowest four non-zero Fourier components to show relative magnitude; (Third Line) the first-order mode shown in blue; (Fourth Line) the second-order mode shown in red; (Fifth Line) the third-order mode shown in green; and (Sixth Line) the fourth-order mode shown in green.

Fourier Analysis of Individual Species in Growing Cell Simulation

The individual species that combine to form the growing domain patterning in Figure 2 are shown separately in Figure S4. As always, the Fourier analysis as shown directly below the corresponding kymograph. As per previous Figures, the first order mode is shown in blue and the second order mode is shown in red.

When the cell is short ($< 2.8\mu m$) all states are stationary (the amplitude of the first and second order modes remains constant). In the stationary regime, the sign of the amplitude of the first order mode differs between the species. MinD (D) and MinE (E_2) in solution as well as the membrane bound MinE homodimer (e_2) all have positive, albeit small, amplitudes of their respective first order modes (blue), while the remaining species (MinD monomer (d), dimer (d_2) and the MinDE complex (d_2e_2)) all have a negative amplitudes.

Once the cell grows to the critical length ($\sim 2.8\mu m$), the patterning begins to oscillate for all species. Visually inspecting Figure S4 in the oscillating regime ($> 2.8\mu m$), it becomes clear that the individual components of the Min system can be broadly clustered into two groups where one group is approximately out of phase with the other.

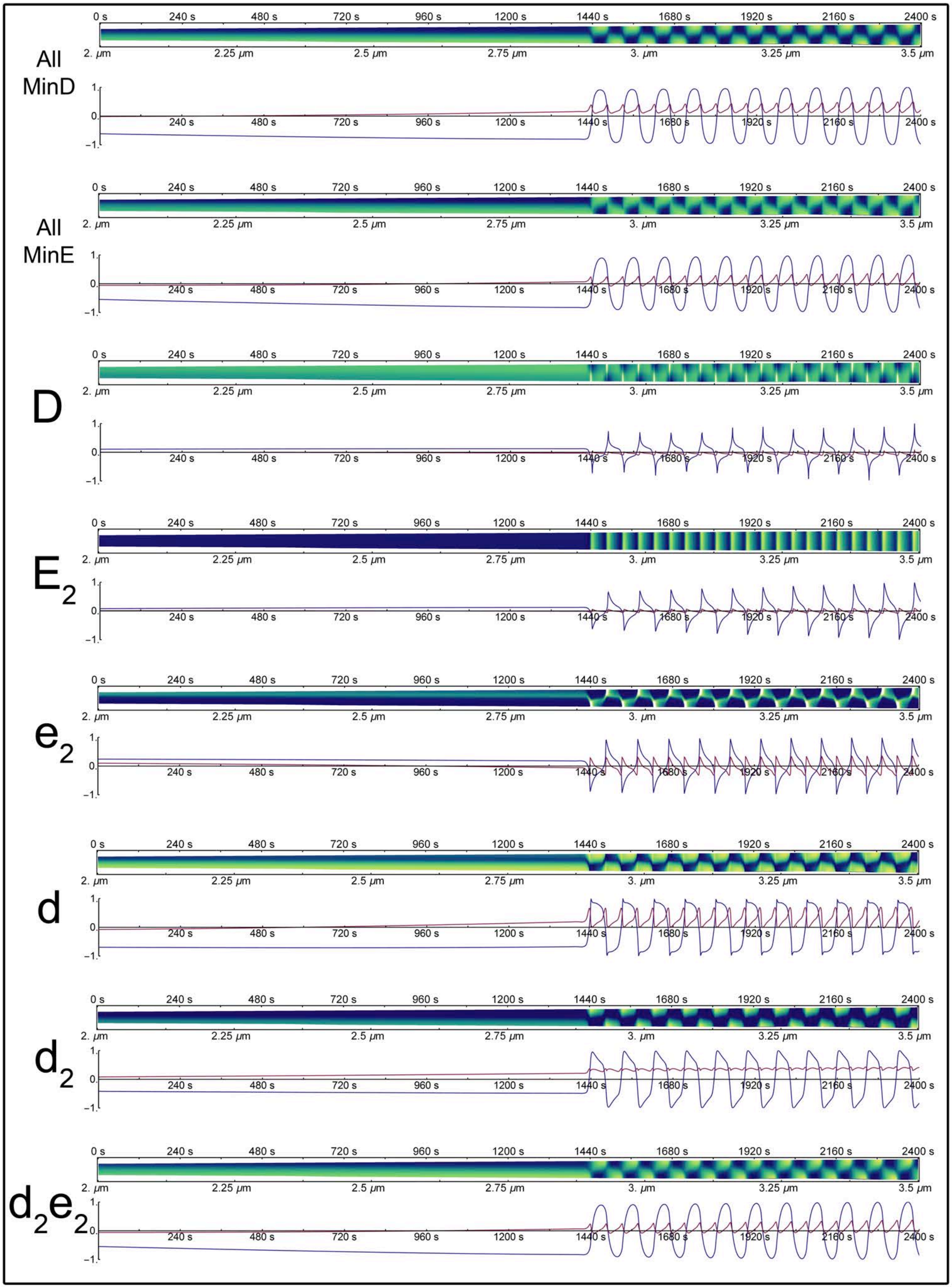

All MinD
All MinE
D
E2
e2
d
d2
d2e2
0 s
240 s
480 s
720 s
960 s
1200 s
1440 s
1680 s
1920 s
2160 s
2400 s
2. μm
2.25 μm
2.5 μm
2.75 μm
3. μm
3.25 μm
3.5 μm
1.
0.
−1.

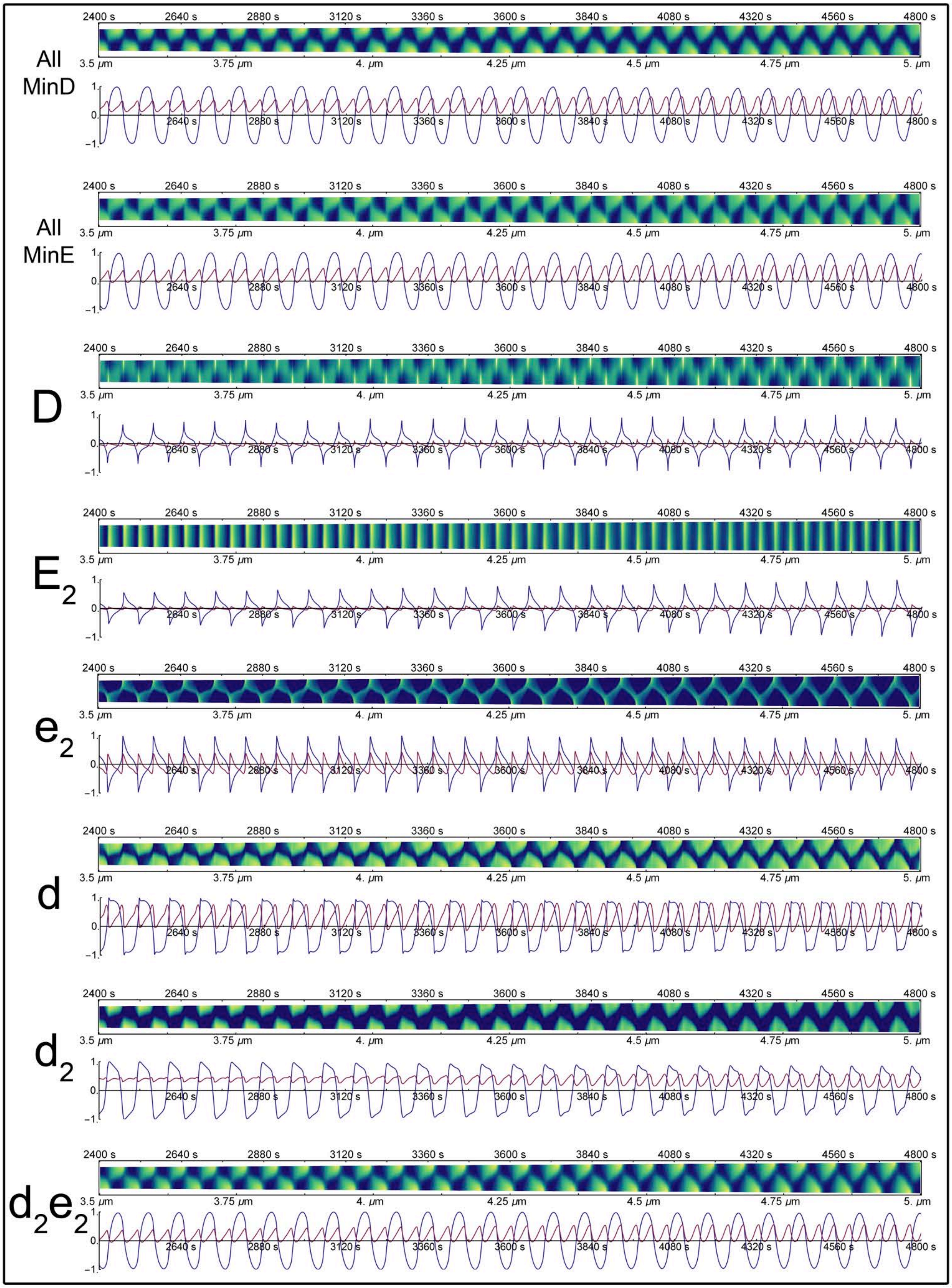
All MinD
All MinE
D
E_2
e_2
d
d_2
d_2e_2
2400 s 2640 s 2880 s 3120 s 3360 s 3600 s 3840 s 4080 s 4320 s 4560 s 4800 s
3.5 μm 3.75 μm 4. μm 4.25 μm 4.5 μm 4.75 μm 5. μm
1. 0. −1.

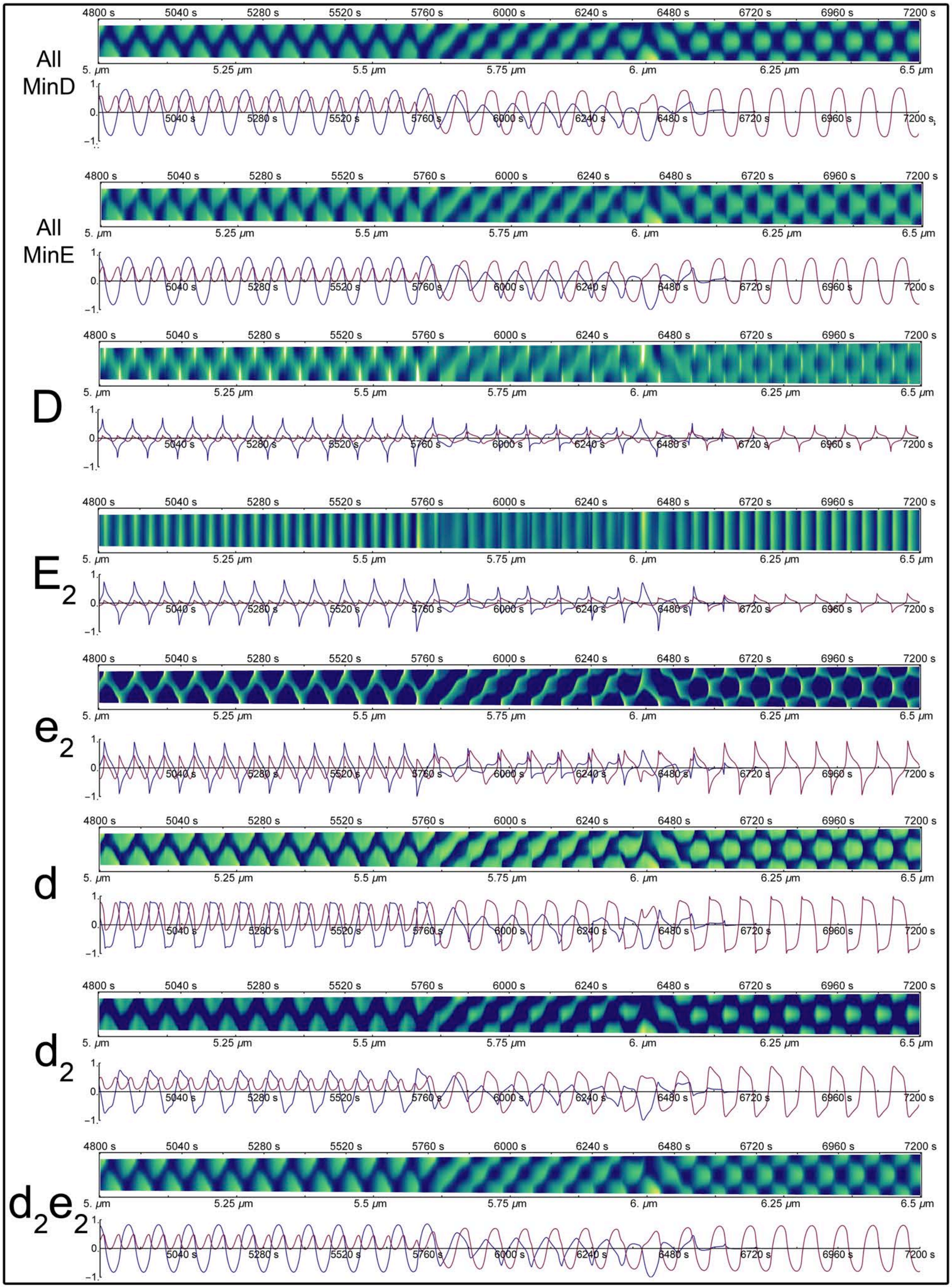

All MinD
All MinE
D
E_2
e_2
d
d_2
d_2e_2
4800 s
5040 s
5280 s
5520 s
5760 s
6000 s
6240 s
6480 s
6720 s
6960 s
7200 s
5. μm
5.25 μm
5.5 μm
5.75 μm
6. μm
6.25 μm
6.5 μm
1.
0.
−1.

Figure S4: Fourier analysis of individual species in growing cell simulation. The individual components of the growing domain simulation shown in Fig 2. For each species the corresponding Fourier analysis is shown beneath each kymograph.

The first order mode of membrane-bound MinD monomer (d), dimer (d_2) and the MinDE (d_2e_2) species are all approximately in phase with each other (they all oscillate from one pole to the other in unison). The remaining species, MinD (D) and MinE (E_2) in solution as well as the membrane bound MinE homodimer (e_2), oscillate together but do so out of phase relative to the other three species. When these species have a maximum at one pole, the other three species will have a maximum at the opposite pole. This grouping of species is the same as that determined by the sign of the amplitude in the stationary regime.

All of the second order mode oscillations are approximately in phase with each other (they all have their respective maximum amplitude at approximately the same time). Given the phase difference between the first order modes, the second order oscillations of three of the second order modes are out of phase with their corresponding first order mode (MinD monomer (d), dimer (d_2) and the MinDE (d_2e_2) species) such that the maximum amplitude of the second order mode lines up with the zero amplitude of the first order mode. The remaining three species (MinD (D) and MinE (E_2) in solution and membrane bound MinE (e_2)) have their second order mode oscillations in phase so that their maximum amplitude lines up the amplitudes of the first order oscillations.

The out of phase second order modes (d , d_2 and d_2e_2) are positively offset while the in phase second order modes (D, E_2 and e_2) oscillate essentially about zero.

To quantify the changes in patterning as the cell grows throughout the oscillating first order patterning regime, Fourier analysis was performed at fixed lengths between $2.8\mu m$ and $4.8\mu m$ in steps of $0.05\mu m$. The results of this analysis are shown in Figure S5.

Figure S5A shows a plot of the average concentration of each species as a function of length. Examining this plot, the concentration of each individual species remains essentially constant throughout the growth of the cell. As a result, changes in the dynamics of the ensemble MinD distribution will come about from changes in the dynamics in the underlying species rather than a simple changing in the relative concentrations of the different species.

Figure S5B shows the change in the first mode amplitude as a function of length for the three species with large amplitudes (d (gold), d_2 (light blue) and d_2e_2 (salmon pink)). While these values change as the cell grows, it is only up to approximately 20% of the original values. In comparison, the change in the first mode amplitude as a function of length for the remaining three species is shown in Figure S5C (D (dark blue), E_2 (red) and e_2 (green)). Here, the amplitude of first order mode oscillation of the two soluble states (D (dark blue), E_2 (red)) increase approximately linearly as the cell grows. So while the amplitude of the membrane-

bound species first order modes remain constant (within 20%), the amplitude of the solution species first order modes increase as the cell length increases.

Figure S5D shows the change in the second order mode amplitudes for the species whose oscillations are centred on zero (D (dark blue), E_2 (red) and e_2 (green)). Similar to the first order mode amplitude (Figure S5C), the amplitude of the second order mode oscillation of the soluble states (D (dark blue), E_2 (red)) monotonically increases with cell length. Interestingly, the second order mode oscillation of the membrane bound MinE homodimer (e_2 (green)) also increases slightly with cell length, while the first order mode amplitude remains constant (Figure 5C).

The amplitude of the second order mode oscillations for the positive offset species are shown in Figure S5E (d (gold), d_2 (light blue) and d_2e_2 (salmon pink)). The amplitude of the second order modes of the MinD monomer (d (gold)) and the MinDE complex (d_2e_2 (salmon pink)) are correlated with both taking similar values, increasing monotonically and increasing less rapidly for lengths greater than $4\mu m$.

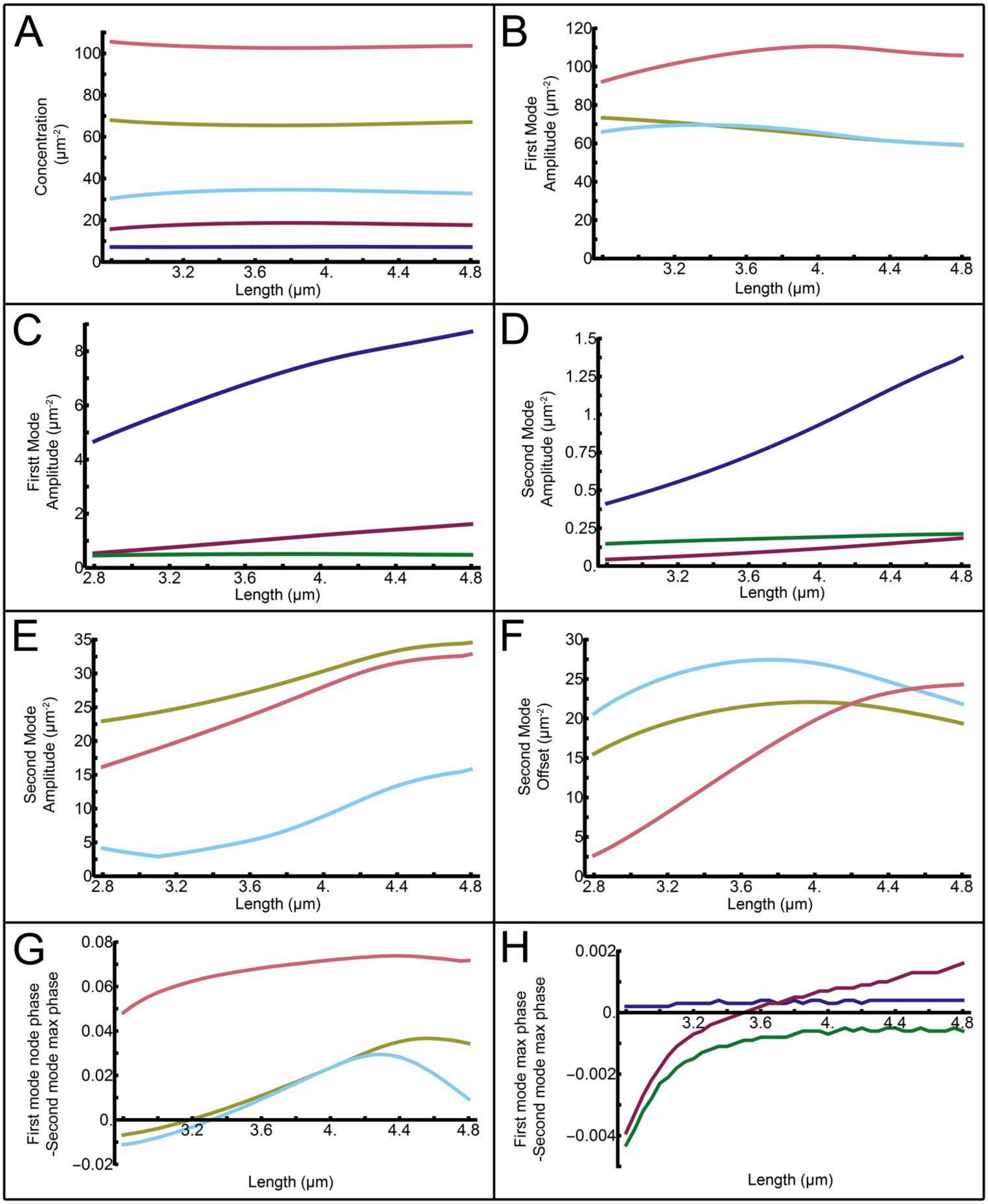
A
Concentration (μm⁻²)
Length (μm)
B
First Mode Amplitude (μm⁻²)
Length (μm)
C
Firstt Mode Amplitude (μm⁻²)
Length (μm)
D
Second Mode Amplitude (μm⁻²)
Length (μm)
E
Second Mode Amplitude (μm⁻²)
Length (μm)
F
Second Mode Offset (μm⁻²)
Length (μm)
G
First mode node phase -Second mode max phase
Length (μm)
H
First mode max phase -Second mode max phase
Length (μm)

Figure S5: Quantitative characterization of individual species in growing cell simulation. The different species are shown in the colours D - dark blue, E_2 -red, e_2 -green, d -gold, d_2 -light blue and d_2e_2 –pink, where upper case letters refer to cytosolic species, while lower case letters refer to membrane-bound species. (A)The change in concentration of each species as a function of cell length. Note that the membrane-bound MinE homodimer (e_2 (green)) occurs at such a low concentration that it is difficult to differentiate from the axis. (B) The change in the amplitude of the first-order mode as a function of length for the three species with small amplitudes (D, E_2 and e_2) (C) As (B) but for the three species with large amplitudes (d, d_2 and d_2e_2) (D) The amplitude of the second-order mode oscillation for the three species whose second-order mode is centred on zero (D, E_2 and e_2) (E) As per (D) but for the three species with positive offset second-order modes (d, d_2 and d_2e_2) (F) The positive offset of the second-order mode as a function of length. Note that this only occurs for the species d, d_2 and d_2e_2. (G) The phase difference between the maximum of the second-order mode and the node of the first-order mode for species whose second-order mode oscillates out of phase (d, d_2 and d_2e_2). (H) The difference in phase between the maxima of the second-order mode and the anti-nodes of the first-order mode for species whose second-order mode oscillates in phase (D, E_2 and e_2).

The changes in the amplitude of the second order mode oscillations for the MinD homodimer (d_2 (light blue)) as a function of length are complicated (Figure S5E). At short lengths ($< 3\mu m$) the amplitude decreases as the length of the cell increases. The amplitude reaches a minimum at a cell length of approximately $3.3\mu m$. As the cell continues to grow, the amplitude increase monotonically. Consistent with the other positively offset modes (d (gold) and d_2e_2 (pink)), the amplitude of the MinD homodimer (d_2 (light blue)) increases less rapidly for cell lengths greater than $4\mu m$ (Figure S5E).

The positive offset of the MinD monomer (d (gold)), dimer (d_2 (light blue)) and the MinDE complex (d_2e_2 (salmon pink))) as a function of length are shown in Figure S5F. The MinD monomer (d (gold)) and dimer (d_2 (light blue)) follow a similar trajectory but are offset from each other. Both increase to a maximum at approximately $3.7\mu m$ before decreasing as the length increases further. The positive offset of the MinDE complex (d_2e_2 (salmon pink))) is reminiscent of its second mode amplitude (Figure S5E), increasing monotonically and increasing less rapidly for lengths greater than $4\mu m$.

As shown Figure S5G, the oscillations of the positive offset second order modes remain out of phase relative to their respective first order mode oscillations. The phase difference between the maximum of the second order modes and the node of the first order mode is maintained below 7.5 % of a period for these species (d (gold)), d_2 (light blue) and d_2e_2 (pink)). Interestingly, for short lengths ($< 3.2\mu m$) the MinD monomer (d (gold)) and dimer (d_2 (light blue)) have the maximum amplitude of their second order mode occur before the amplitude of the first order

mode is zero. This is observed as the negative values for the monomer (d (gold)) and dimer (d_2 (light blue)) species in Figure S5G. However, as the cell length increases, the reverse then becomes true, where the amplitude of the first order mode is zero then the maximum amplitude of the second order mode occurs. For the MinDE complex (d_2e_2 (pink))) the amplitude of the first order mode becoming zero always occurs first.

Figure S5H shows that while some small change occurs, the species that oscillate in phase with their first order mode (D (dark blue), E_2 (red) and e_2 (green)) remain very strongly phase locked at all cell lengths. The difference in phase between the maxima of the second order mode and the anti-nodes of the first order mode is always less than 0.4% of a period.
